# Determination of Chewing Count from Video Recordings Using Discrete Wavelet Decomposition and Low Pass Filtration

**DOI:** 10.3390/s21206806

**Published:** 2021-10-13

**Authors:** Sana Alshboul, Mohammad Fraiwan

**Affiliations:** Department of Computer Engineering, Jordan University of Science and Technology, P.O. Box 3030, Irbid 22110, Jordan; smalshboul16@cit.just.edu.jo

**Keywords:** chewing, smart devices, discrete wavelet decomposition, low pass filter, number of chews

## Abstract

Several studies have shown the importance of proper chewing and the effect of chewing speed on the human health in terms of caloric intake and even cognitive functions. This study aims at designing algorithms for determining the chew count from video recordings of subjects consuming food items. A novel algorithm based on image and signal processing techniques has been developed to continuously capture the area of interest from the video clips, determine facial landmarks, generate the chewing signal, and process the signal with two methods: low pass filter, and discrete wavelet decomposition. Peak detection was used to determine the chew count from the output of the processed chewing signal. The system was tested using recordings from 100 subjects at three different chewing speeds (i.e., slow, normal, and fast) without any constraints on gender, skin color, facial hair, or ambience. The low pass filter algorithm achieved the best mean absolute percentage error of 6.48%, 7.76%, and 8.38% for the slow, normal, and fast chewing speeds, respectively. The performance was also evaluated using the Bland-Altman plot, which showed that most of the points lie within the lines of agreement. However, the algorithm needs improvement for faster chewing, but it surpasses the performance of the relevant literature. This research provides a reliable and accurate method for determining the chew count. The proposed methods facilitate the study of the chewing behavior in natural settings without any cumbersome hardware that may affect the results. This work can facilitate research into chewing behavior while using smart devices.

## 1. Introduction

Chewing (i.e., mastication) is the action of crushing and grounding food by the teeth. It is an important process that represents the first step of digestion by which the surface area of the food is increased to allow for easy swallowing and efficient breakdown by enzymes. Healthy nutrition is affected by several factors related to chewing, including; food intake, chewing behavior, chewing time, chewing speed and the bolus size.

Monitoring and study of the chewing process is important. Abnormal chewing behavior could be an indication of some ailments (e.g., anorexia, tooth decay, etc.), which may reduce the chewing speed or the bolus size. Moreover, people suffering from binge eating disorder tend to consume large amounts of food in a short time and are subject to greater risk of high blood pressure and cardiovascular diseases [1]. In addition, some researchers attempted to establish calibrated model for the caloric intake based on the number of bites and chew count [2]. Thus, there is a need to establish automated portable methods for the correct determination of the chew count [3]. Also, eating while using mobile handheld devices is becoming common with children. This phenomenon has a great effect on eating habits, which in turn influence the health of individuals (e.g., obesity and overweight). Recent research suggests that children who use electronics for longer hours or eat while using those devices have higher Body Mass Index (BMI) [4].

Manually counting chews by trained clinicians and the effort involved in studies enlisting even small number of subjects is large considering the number of chews per minute. The process is tedious, time consuming, and error prone. The objective of this paper is to automatically determine the chew count from video recordings of subject munching on food while using camera-equipped electronic devices. This research develops a method to automatically count the number of chews appearing in the video recording. The results from this work can facilitate greater research in chewing behavior and its relationship with human health. The contributions of this paper are as follows:

- We record chewing video data from 100 subjects at three speeds (slow, normal, and fast).
- We use image processing techniques to isolate and extract the videos of the subject’s face away from artifacts.
- We extract signals corresponding to the various movements during the chewing action.
- We propose two algorithms to count the number of chews automatically based on Discrete Wavelet Decomposition and low pass filters.
- We achieve a low mean percentage error in automatically counting the number of chews.

The remainder of this paper is organized as follows: In Section 2 we provide a background into the chewing process and its health ramifications, and the related literature in automatic chew counting. Section 3 describes in detail the data collection process and the proposed methods for determining the chew count. Performance evaluation metrics and the corresponding results are reported in Section 4. This is followed by a discussion in Section 5 of the advantages and limitations of the reported work. The conclusion and future work are presented in Section 6.

## 2. Background and Related Work

Chewing is the process of grinding a large piece of food between the teeth to convert the food to small bolus that could be swallowed [5,6]. Recently, chewing behavior is considered one factor associated with increased risk of diseases such as obesity and diabetes, which may result from abnormal chewing behavior or from eating disorders [7]. Changes to chewing behavior may be attributed to social and economic factors that may affect food intake and food selection. For example, consuming food while driving or during the usage of smart devices may lead to fast food intake and a reduction in mealtime [7]. In the next subsection, we discuss the importance of investigating chewing behavior. Such literature signifies the importance and real-life applications of the automated count of chews. After that, we analyze the related works and their shortcomings.

### 2.1. Chewing and Health

The relationship between chewing behavior and various health aspects is continuously being investigated in the literature. [8] showed that eating slowly might reduce the risks of overweight and underweight in Japanese preschoolers. This was corroborated by the results of [9], wherein obese subjects had lower number of chews per gram of food in comparison to a subject having normal weight. In this regard, relevant literature has shown that increasing the chew count by 150–200% may reduce the food mass intake by up to 15% [10]. Similarly, other studies have shown that prolonged chewing before swallowing may lead to lower caloric intake [11,12].

Chewing has also been found to be beneficial to brain functions. Chen et al. [13] showed that chewing is an effective activity for maintaining the part of the nervous systems responsible for spatial memory and learning (i.e., the hippocampus). Preserving the hippocampus can reduce brain deterioration with age. Chuhuaicura et al. [14] supported the hypothesis of the correlation between mastication and cognitive protection, and they identified seven areas in the brain prefrontal cortex that could be affected by increasing the mastication [15]. In general, mastication plays as a protection factor from cognitive deterioration and neurodegenerative diseases [13,15,16].

### 2.2. Automatic Chew Counting

Traditional methods used for determining the chew count were either manual or automatic (i.e., using pervasive hardware) [17]. Manual methods are inherently tedious, prone to errors, and un-scalable to large number of subjects. They rely on inspecting visual recordings or direct viewing of subjects. For example, Moraru et al. [18] used visual observation to collect chewing count data from 34 subjects. Other studies [2,12] used similar approach.

Automated methods employ a range of devices that vary in sophistication and cost. Some studies used Electromyography (EMG) to record the chew count of a small number of subjects (i.e., less than 10), which is understood given that special electrodes, EMG device, and professional help are required to perform the recording [19,20,21]. In another study, piezoelectric and printed strain sensors were used in characterizing the chewing behavior of five subjects [22]. However, their approach relied on the subjects to report their own chewing behavior via a push button. Such an approach may be biased as the subjects positively influenced the quality of the input signal (i.e., the chewing behavior was unnatural). Nonetheless, the reported mean absolute error was 8% even with such input. Similarly, Fontana et al. [2] employed the same input method. They used the annotated data to train an artificial neural networks model (ANN) and their research achieved a mean absolute error of 15.01%. Amft et al. [23] proposed counting chews using sound analysis of audio recordings of the chewing process. However, such a method differs among subjects and may be prone to ambient and other types of noise especially if the subject is using an electronic device (e.g., playing multimedia) while eating. Nonetheless, noise-resilient algorithms for chewing detection were proposed by Bedri et al. [24] using a combination of acoustic, optical, and inertial sensors. They achieved an accuracy of 93% and an F1-score of 80.1% in unconstrained free living evaluation. Similarly, Papapanagiotou et al. [25] used convolutional neural networks to achieve a 98% accuracy and F1-score of 88.3%. Recently, Hossain et al. [26] used a similar approach to detect faces, which they followed by transfer learning using AlexNet to classify images as bite or not, and used affine optical flow to detection rotational movement in the detect faces. They reported a mean accuracy of 88.9±7.4% for chew count. However, deep learning algorithms are known to be slow and consume significant resources.

In general, hardware-based methods may cause discomfort to child subjects and incur high cost in large-scale experiments. Additionally, remote or at a distance studies may not be possible if special procedures are required to fit the hardware. Cadavid et al. [27] used an active appearance model (AAM) to detect chewing events from captured images of the subject’s face. They noticed that the AMM parameters displayed periodic variations in response to the chewing behavior, which were different from other facial activities (e.g., talking). Thus, spectral analysis was used to derive features for a support vector machine classification model. The dimensionality of the features was reduced using principle component analysis in order to reduce the system overhead. However, their approach requires extensive space and computational overhead [28]. They achieved an accuracy of 93%, but that was accomplished using leave one subject out validation, which is not recommended for their small dataset (i.e., 37 subjects) [29].

## 3. Material and Methods

### 3.1. Ethical Approvals

The current study was approved by the institutional review board (IRB No. 29/11/2018) at King Abdullah University Hospital (KAUH) and the Deanship of Scientific Research at Jordan University of Science and Technology in Jordan.

### 3.2. Procedure

Written informed consent was sought and provided prior to the study commencement. For underage subjects, their parents filled the consent form, which needed to be signed if they voluntarily accepted their child’s participation. The research assistants received intensive training by the lead investigators on the data collection process, as well as the data entry. The information package included an information sheet describing the study purpose and procedure in details, the consent form (including consent to publication of images), and a parental/self-reporting questionnaire that contains demographics and other relevant information.

### 3.3. Participants

The current study enrolled 100 randomly selected subjects. A total of 375 information packages were randomly distributed prior to data collection. Of those, 275 (73.3%) recipients refused to participate. The subjects included a mix of children and adults, with an age range of 6–76 years (mean = 19.72, standard deviation = 11.03). Fifty-six of the subjects were children and 44 were adults, and 58 were males. There were no restrictions regarding skin color, facial hair, hairstyle, head cover, or wearing glasses (medical or otherwise).

### 3.4. Data Collection

A Huawei Y7 Prime 2018 smartphone main camera was used for video recording. It is a 13 MP camera with 1080p@30fps resolution. The subjects were asked to face the camera and eat a crunchy food sample (e.g., cucumber). Each subject recorded three one-minute clips corresponding to three speeds (i.e., slow, normal, and fast). There was no specific environment for the dataset collection, and no additional constrains were set during video recording. Videos were recorded in a variety of setups (i.e., outdoors, indoors in a room, and in public places) and with different light intensities.

Objective reference is required as a gold standard for performance evaluation. To this end, three annotators were trained by the principle investigators to count the number of chews in video recordings, and the training videos were not included in the dataset. Each annotator worked independently from all others and recorded the number of chews in each of the 300 video clips (i.e., 100 subjects with 3 recordings each). The annotators were allowed to pause and rewind the videos for accurate counting.

Upon completing the annotation, the reliability of the process was verified using Intra-class correlation coefficient (ICC) [30]. Table 1 shows the ICC values for all annotators as well as pair wise comparisons among them. The lowest value in the table is 0.83 between annotators 2 and 3, which is considered an excellent value [31].

### 3.5. Determining the Chew Count

**Figure 1:**
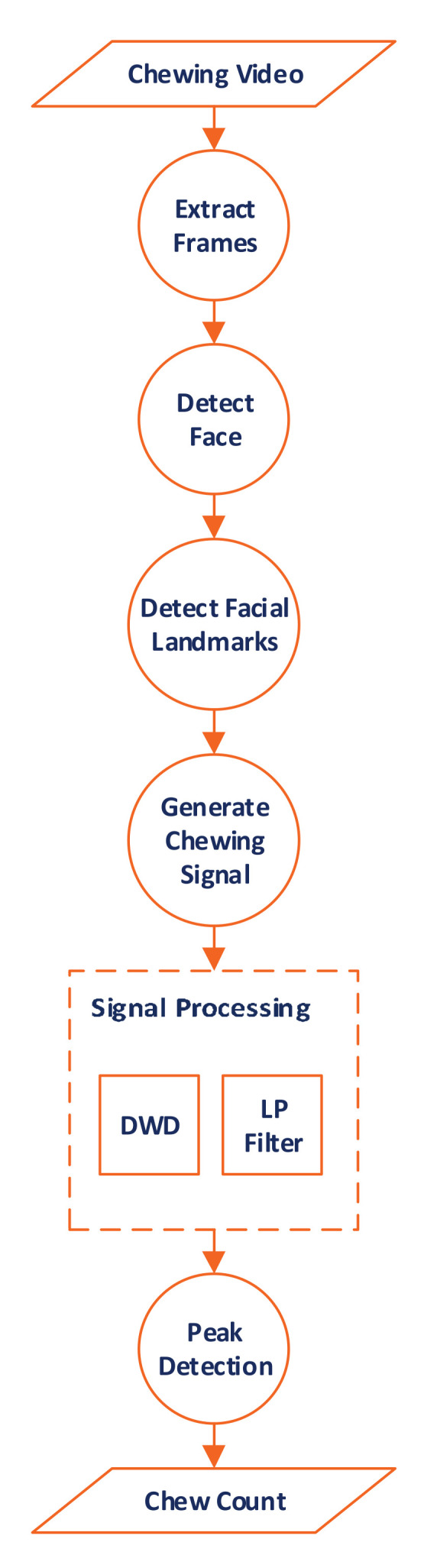
The general steps to count the number of chews from the input video clip.

Figure 1 shows the general steps taken to count the number of chews. Given a video recording of the subject while eating, the algorithm works by first extracting individual frames as separate images. In each image, the face of the subject is identified using the Viola-Jones algorithm [32] (Section 3.5.1). However, not all of the face is of interest to chew counting, only a few landmarks, which are indicators of mastication, are important. Thus, the Kasemi and Sullivan landmark detector [33] was employed to detect facial landmarks (Section 3.5.2). The Euclidean distance between a reference point and each of the identified facial landmarks is measured and the average is calculated. Since chewing involves jaw motion, there is a need to treat successive Euclidean distance averages as time series data generated using the mean Euclidean distance from each video frame, which results in the chewing signal (Section 3.5.3). After that, filtering techniques employing LPF or DWD retrain the relevant frequencies (Section 3.5.4). Finally, a peak counting determines the number of chews excluding biting peaks (Section 3.5.5). In the next few subsections, we will go through each one of the steps in detail. These steps were implemented using Matlab 2020a software.

#### 3.5.1. Face Detection

**Figure 2:**
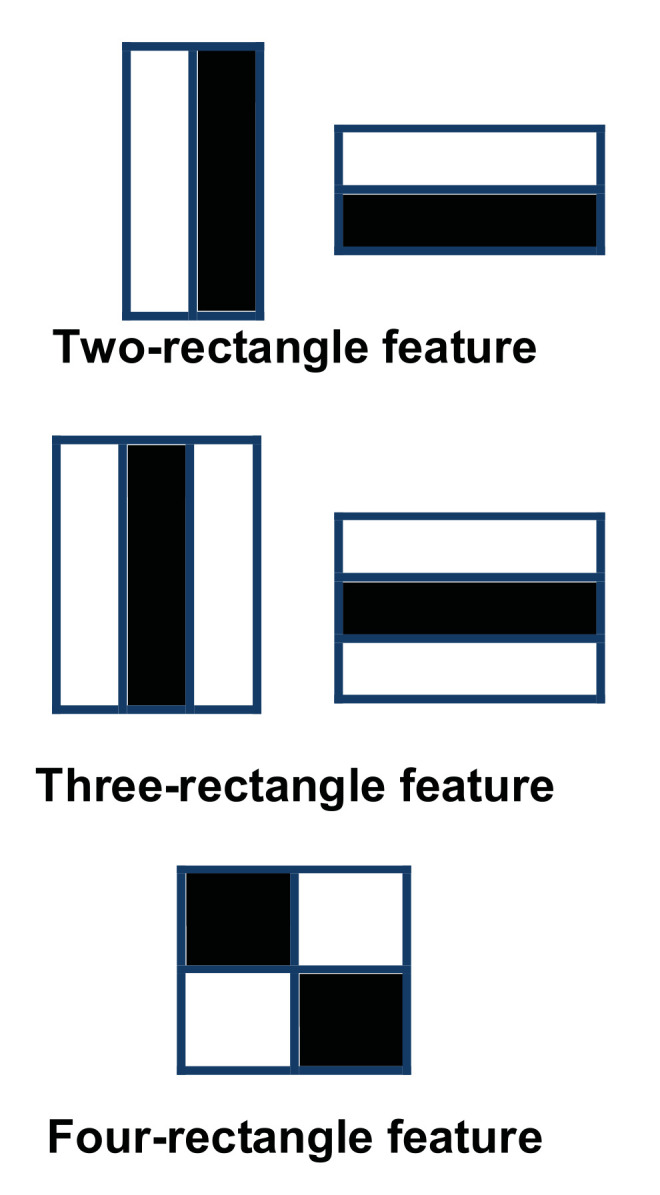
Haar rectangular features.

**Figure 3:**
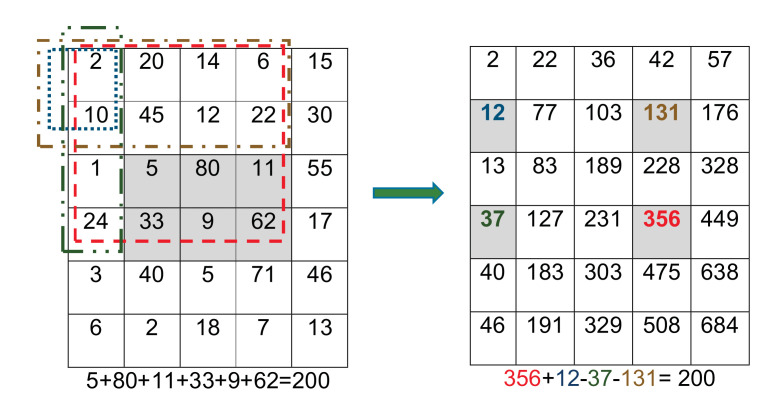
The intensities in the original image (**left**) and the corresponding integral image (**right**). Calculating the intensity of the shaded box requires only four indices in the integral image regardless of the number of pixels in the box.

The first step in the algorithm aims to detect the face of the subject. To this end, the Viola-Jones face detector was employed. The algorithm was chosen because it is fast and has high detection accuracy [32]. It works in the following steps:

1. The image is converted to gray scale, which reduces the overhead. However, once the face is detected, the location is marked in the colored image.
2. The image is scanned to search for intensity differences that may represent facial features. This is done using boxes called Haar rectangles [34].These boxes are moved so that every tile in the image is covered. Figure 2 shows a set of three Haar features (HFs); two-rectangle, three-rectangle, and four-rectangle. These features represent regions with different shades in an image. For example, the eyebrows will appear darker in comparison to the surrounding skin. Similarly, the top of the nose may seem brighter than the sides.
3. Each box is represented by a matrix of values corresponding to the pixel color intensities in that box. The darker the pixel the closer the corresponding value to 1. A Feature is generated by the difference between the sum of pixel values in the dark region and the sum of pixel values in the light region.
4. The previous calculations can cause high computational overhead because of the large number of pixels. Therefore, the process is adjusted to use an integral image (i.e., a summed-area table). Each value, l(x,y) , in the integral image is the summation of all pixel values that lie above and to the left of (x,y) in the original image inclusively, see Equation (1). Figure 3 shows an example matrix representing the original image and the corresponding integral image. Using the integral image, calculating the intensities of any rectangular area of any size in the original image requires four values only. Moreover, the integral image is calculated with a single pass over all pixels. This method greatly improves the efficiency of calculating the Haar feature rectangles.
5. Scanning the image using the rectangular boxes will generate a set of intensity values, which form the input to the classification process. The output of this step indicates whether or not a feature is likely to be part of the face. The Viola-Jones algorithm uses adaptive boosting (AdaBoost), which employs a weak learner constraint to select few features out of thousands of possible features. The algorithm training dataset contained 4960 annotated facial images as well as 9544 other images without faces [32].
6. Cascaded or ensemble classification. This step further refines the classification process by attempting to discard the background regions by increasing the complexity of classifiers in cascade. The collective effect of the weak classifiers selects the best combination of features and their associated weights.
(1) $$l\left(x,y\right)=\sum_{x^{\prime }\leq x,y^{\prime }\leq y}v\left(x^{\prime },y^{\prime }\right),$$
  where v(x’,y’) is the value of the pixel at (x’,y’) .

#### 3.5.2. Facial Landmarks Detection

The Viola-Jones algorithm generates a bounding box around the face of the subject. However, the face as a whole is not useful by itself for chew counting. Thus, Kasemi and Sullivan landmark detector [33] was employed to identify key facial features and their location on the face. The facial landmark detector estimates the position of the facial landmarks using an ensemble of regression trees (ERT) based on sparse pixel set intensities, which are used as an input to the regressors. The pixel intensities are selected using a gradient boosting algorithm and a prior probability of the distance between pairs of input pixels. The face image is transformed into an initial shape and the features are extracted to update the current shape vector. This procedure is repeated several times until convergence is reached. After that, intensities of the sparse pixels are indexed on the initial shape. Each regressor estimates the current shape from an initial shape estimation to solve the problem of face alignment. The initial shape can be selected by the mean shape of the centered and scaled face image.

This procedure results in a 192×2 vector representing the (x,y) coordinates of 192 points on the subject’s face. However, such number of facial points is excessive, redundant, and consumes large space and processing power. The determination of the facial landmarks forms the basis for the identification of the chewing motion. Several useful observations were drawn from analyzing the chewing process, as follows:

1. The lower lip moves up and down during crushing the bolus in between the upper and lower jaws. Furthermore, the lower lip moves slightly to the left and right during the bolus motion in the mouth, but the motion of the lower lip decreases when the subject swallows. Moreover, the lower lip motion is undiscernible when the chewing speed is too slow and when the food texture is neither solid nor crispy. In addition, the separation between the two lips increases when the subject is taking a bite.
2. The upper lip motion is unbeneficial for counting chews as it is undiscernible across video frames. This mainly due to its connection to the immobile maxilla.
3. The corner points on the edge of the mouth move in an oval trajectory, which could be a result of smiling or other facial expressions. Thus, they were ignored.

**Figure 4:**
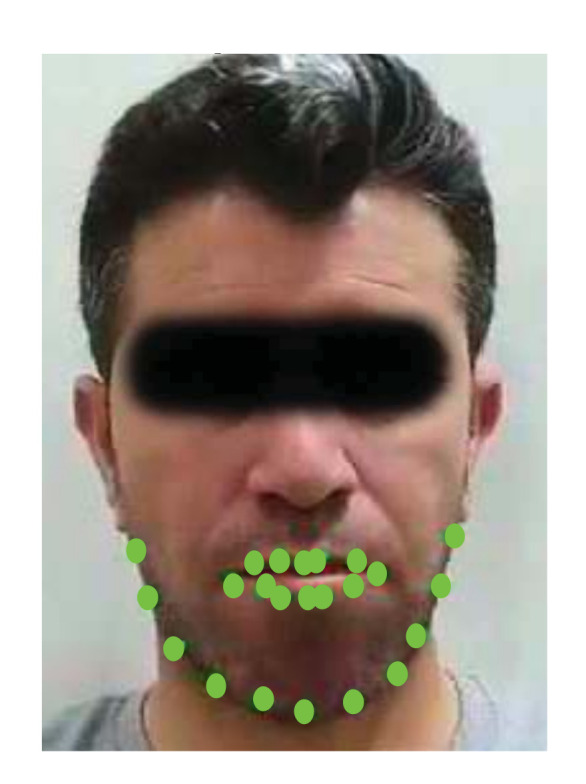
Facial landmark detection showing the 11 jaw and 15 mouth landmarks. Only the 11 jaw landmarks were used in counting chews.

Careful inspection of the chewing process revealed that most of the points responding to the chewing operation are located in the chin and jawline regions. Therefore, only 11 points in the chin and jawline were used, see Figure 4. They displayed consistency and a stable chewing pattern during chewing regardless of the speed. Moreover, the motion is immune to facial expressions (e.g., smiling). In addition, the points are visible during food intake. Thus, the motion of the jawline points was used for counting purposes. These points move in three ways, as follows:

1. Up and down during for crushing/chewing the food.
2. Sideways during bolus motion across the mouth sides.
3. A large downward movement for every food bite.

#### 3.5.3. Generation of the Chewing Signal

We define the up down mandible motion as one chew. To measure this motion, a reference point was required with the constraint that it is unaffected by the chewing motion, random movement, and may not be hidden during chewing. To this end, the upper left corner of the face bounding box was chosen as a reference for all movements. This box tracks the face throughout the recording and represents a fixed reference frame for the jawline points. The Euclidean distance (ED) was measured for each frame between every jawline point (*x*, *y*) and the reference point (u,v) , and the average was taken for the 11 points, see Equation (2).

(2) $$ED=\frac{1}{11}\sum_{p=1}^{p=11}\sqrt{{\left(x-u\right)}^{2}+{\left(y-v\right)}^{2}}$$

**Figure 5:**
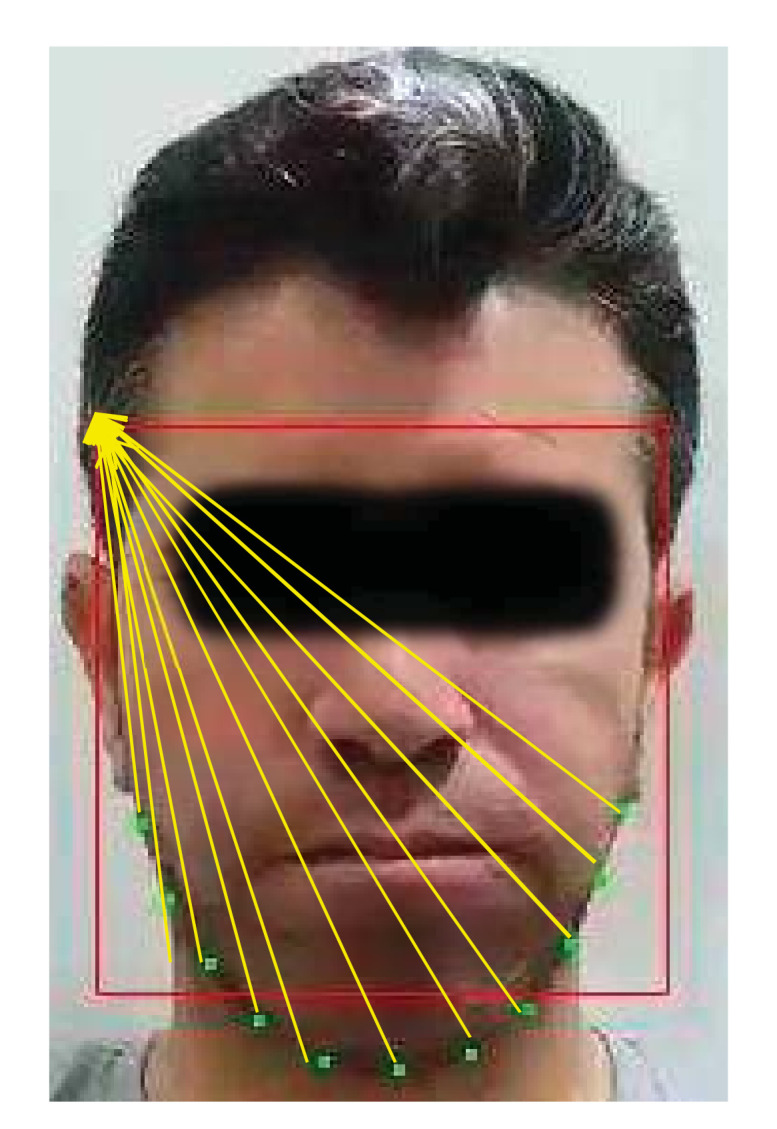
The Euclidean distance between chin/jaw landmarks and the upper left corner of the face rectangle.

**Figure 6:**
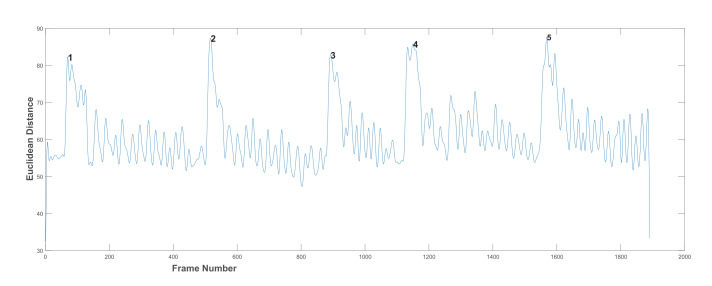
The chewing signal and five biting peaks.

Figure 5 shows an example of the ED as measured between the reference point and the jawline points used for counting chews. The ED values measured throughout the duration of the chewing clip form a signal that represents the chewing pattern, see Figure 6. The labelled peaks in Figure 6 represent the subject taking a bite and they were discounted from the total chew count. Moreover, the signal inherently contains some noise due to the subject’s movement and swallowing. For example, the sideways movement of the head. Therefore, signal processing techniques were required to correctly identify the patterns resulting from the actual chewing.

#### 3.5.4. Chewing Signal Processing

**Figure 7:**
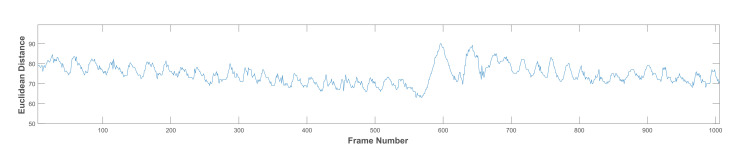
A chewing signal with many fake peaks caused by noise.

**Figure 8:**
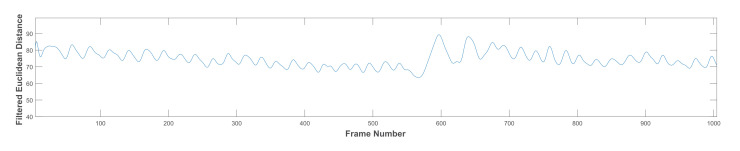
The same signal in Figure 7 after low pass filtration.

**Figure 9:**
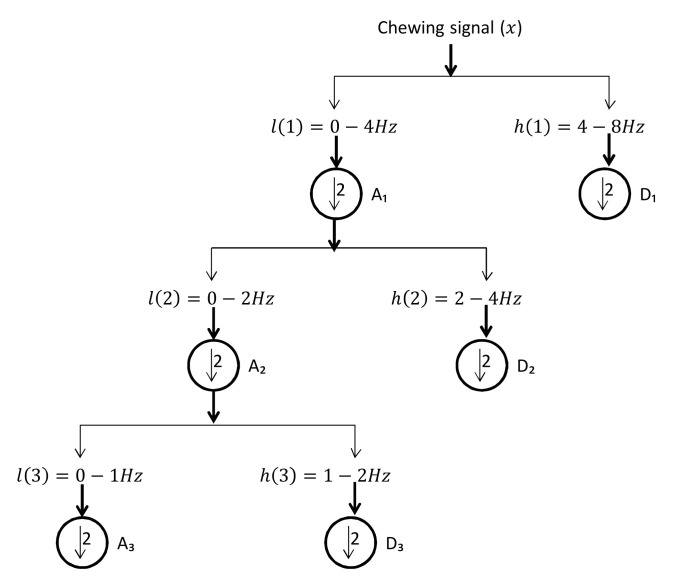
Three-level discrete wavelet decomposition.

**Figure 10:**
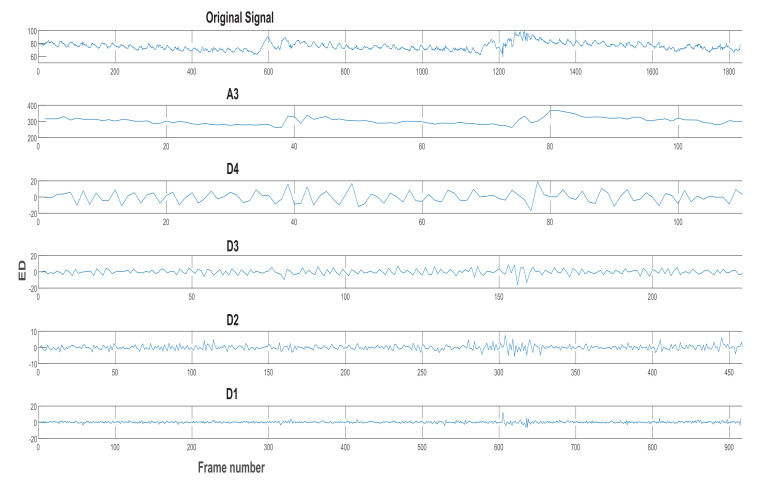
Four-level DWD of the signal in Figure 7.

As previously stated, the chewing signal carries some noise due to the subject’s movement, mandible motion, and other artefacts (e.g., variations in the head bounding box). We experiment with two signal processing methods to improve the signal usefulness, as follows:

- Low pass filter (LPF): a LPF was designed with a cut-off frequency of 1 Hz and a sampling rate of 30 Hz [35]. It is a linear phase minimum order finite impulse response filter. The measured frequencies in the collected dataset ranged between 0.4 and 2.3 Hz for all chewing speeds. However, some of these frequencies resulted from variations in the mandible motion before the completion of one chew. Thus, the frequencies that are not representing actual chewing were removed. This was accomplished by assigning a proper passband frequency. Several passband frequencies and sampling rates were tested, and a 1 Hz passband frequency and 50 Hz sampling rate achieved the best results. Figure 7 shows the original signal with many fake peaks caused by noise. Whereas Figure 8 shows the smoothing of the signal and the elimination of most of these peaks after LPF application.
- Discrete wavelet decomposition (DWD): DWD is a discrete version of the continuous wavelet transform [36]. It retains the important features and reduces the computational complexity in comparison to the continuous wavelet transform [37]. In DWD, the signal is decomposed using low and high pass filters into approximation (A) and detail (D) coefficients, respectively. Further reduction to the frequency was achieved by applying the same procedure to the resulting approximation coefficients. A Daubechies mother wavelet with tab equal 4 was used, which achieve the best smoothing effect while retaining the important features. The sampling rate in the chewing signal was 30 Hz and the chewing signal frequency was 0–16 Hz, because of the noise in the signal that comes from the unwanted movements and from the fast chewing speed videos. Thus, three levels of decomposition were required to reach the closest frequency of chewing (i.e., 1–2 Hz) for normal speed, see Figure 9. This corresponds to 1 to 2 chews per second. The frequency resolution can be increased/decreased to match the chewing speed and the associated chewing signal frequency, see Figure 10.

#### 3.5.5. Counting Chews

The output from either one of the two signal processing techniques (i.e., LPF and DWS) forms the basis for determining the number of chews. A peak detection algorithm was employed to detect the chewing markers. The algorithm works by finding every local maximum in the signal that is larger than the adjacent two neighboring points, where every peak represents one chew. The Minimum-Peak-Height (MPH) parameter for peak detection was set for LPF to half the average of all peak heights (PH), see Equation (3). For DWD and slow chewing videos, the MPH was set to half the average of PH see Equation (4). Equations (5) and (6) show the values of the MPH for the DWD processing of the normal and fast chewing speeds.

**Figure 11:**
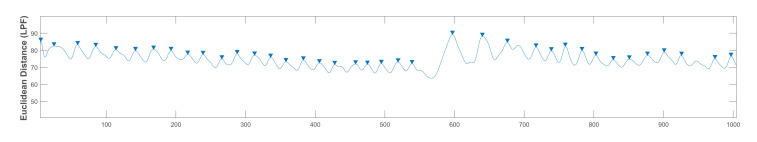
LPF output for counting chewing peaks in the processed signal.

**Figure 12:**
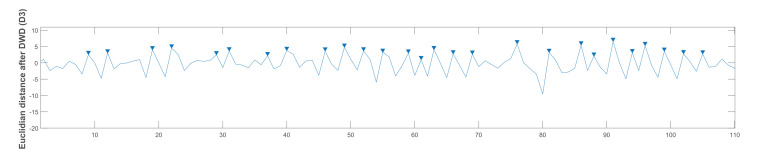
DWD output for counting chewing peaks in the processed signal.

The MPH was set differently for the three chewing speed signals because it was observed that the mandible movement changes in response to different chewing speeds. The highest displacement occurred in the slow chewing speed signals. Thus, the chewing peaks were high in comparison to false peaks (i.e., noise). On the other hand, the mandible displacement was small in the fast chewing speed signals, so more of the peaks need to be counted. Figure 11 shows the application of the peak counting algorithm on the LPF-processed signal, and Figure 12 shows the results from the DWD output.

(3) $$MPH_{LPF}=\frac{1}{2}\times \frac{1}{n}\sum_{i=0}^{n}P_{H}$$

(4) $$MPH_{DWD\_slow}=\frac{1}{2}\times \frac{1}{n}\sum_{i=0}^{n}P_{H}$$

(5) $$MPH_{DWD\_normal}=\frac{1}{3}\times \frac{1}{n}\sum_{i=0}^{n}P_{H}$$

(6) $$MPH_{DWD\_fast}=\frac{1}{4}\times \frac{1}{n}\sum_{i=0}^{n}P_{H}$$

## 4. Results and Evaluation

### 4.1. Complexity Analysis

As presented earlier, the proposed work relies on software-based methods as opposed to hardware solutions (i.e., dedicated sensors). Sensing and counting hardware maybe invasive but it provides less computationally intensive option. However, the approach used in this paper is based upon well-established practical methods with linear time complexity. The Viola-Jones face detector runs in linear time O(N) , where *N* is the number of pixels in the image. The calculations are done within a small region of interest in the integral image. Moreover, the Haar features are computed in constant time [38]. The next step is facial landmark detection, which uses the Kazemi and Sullivan [33]. Both this and the Viola-Jones algorithms are considered real-time algorithms with low complexity and high speed [39]. The third step computes the average Euclidean distance for 11 chin/jaw landmarks in each frame. At a frame rate of 30 fps, this computation is negligible. Next, the chewing signal is filtered using either LPF or DWD, with the later having linear time complexity [40]. The last step is counting peaks, which inspects the elements before and after each possible peak. Thus, it requires linear number of steps.

### 4.2. Performance Evaluation Metrics

The performance of the proposed methods was evaluated in terms of the absolute error ( AE ), mean absolute percentage error ( MAPE ), and root mean squared error ( RMSE ). Each one of these metrics provides a different insight into the accuracy of the counting algorithm. RMSE tends to penalize large errors. On the other hand, AE and MAPE are easier to interpret. In addition, MAPE allows comparisons between varying chewing counts as the error is relative to the gold standard. Equations (7)–(9) to show the formulas for calculating these metrics.

(7) $$AE=|Actual_{count}-Measured_{count}|$$

(8) $$MAPE=\frac{1}{n}\sum_{1}^{n}\frac{|Actual_{count}-Measured_{count}|}{Actual_{count}}\times 100\%$$

(9) $$RMSE=\sqrt{\frac{1}{n}\sum_{1}^{n}{\left(Actual_{count}-Measured_{count}\right)}^{2}}$$

The Bland-Altman plot was used to measure the agreement between the proposed algorithms and the actual chew count as determine by each annotator. This is a graphical method that plots the difference between the calculated values and the gold standard values against the average of the two methods. Any two methods can be used interchangeably used if 95% of the data points are located within the limits of agreement, which are defined as the mean ±1.96×SD [41].

### 4.3. Results

Table 2 shows the AE for the two signal processing methods. The average AE is lowest for the slow chewing speed for both LPF and DWD, although LP slightly outperforms DWD with an AE of 5.42±4.61 . Moreover, the error is higher for faster speeds. The same trend appears in Table 3 and Table 4 for MAPE and RMSE respectively. Again, LPF achieved superior performance for normal chewing with 7.76% and 7.93 for MAPE and RMSE , respectively.

**Figure 13:**
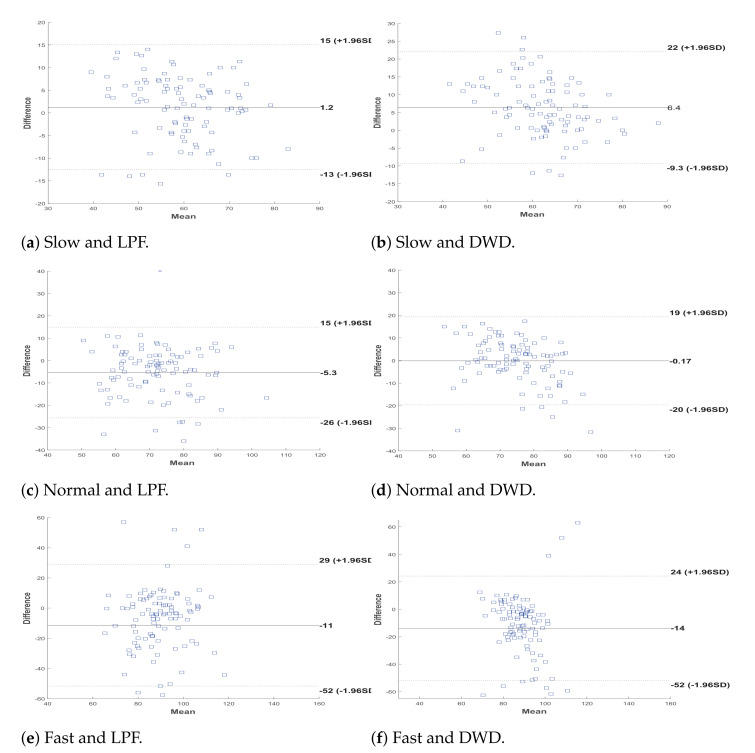
Bland-Altman plots for the chewing counts at the three speeds with LPF and DWD processing.

Figure 13 show the Bland-Altman plot for the agreement between the proposed algorithm and the average of the three annotators (i.e., the gold standard) using LPF or DWD. The figures show that most of the points are within the lines of agreement. However, the algorithm needs improvement for faster chewing. Nonetheless, our method can be used interchangeably with the manual measuring techniques but provides the advantages of automated measurement and reliable results. This serves as an evidence of the accuracy and efficacy of the proposed approach.

Table 5 shows a comparison to the related literature in terms of best average error, the counting method, and the number of subjects recruited by the researchers. The evaluation of the proposed approach in this paper is based on the largest number of subjects and achieved the least average error. Almost all of these approaches rely on dedicated hardware or signals extracted from this hardware. On the other hand, our work uses input from camera-equipped smart devices. Moreover, the number of subject recruited in most studies is small, which may result in overfitting of the proposed methods to the specific chewing pattern. Additionally, these studies did not test for different chewing speeds although multiple food types were used to record chewing cycles.

## 5. Discussion

The work in this paper presents a method for the automatic counting of chewing from video recordings. The results from both the LPF and DWD approaches suggest that the proposed method can be used as an objective and accurate chewing counter. In comparison to the literature, the method was tested on a reasonably large number of subjects and chewing speeds.

In both signal processing techniques, the algorithm was used to estimate chew counts in manually annotated chewing clips and was able to achieve a best AE, MAPE, and RMS of 5.42±4.61 , 6.48%, and 5.56, respectively. However, this was achieved for slow chewing speeds. The same values for the normal chewing were 7.47±6.85 , 7.76%, and 7.93, respectively. Moreover, given that the human counting accuracy is typically 5.7%±11.2% [3], our results present an excellent objective and automated methodology for accurate chew counting. In addition, the results in Figure 11 show that the difference between the measured and annotated values to fall in the region over the mean, which may be explained by the tendency of the annotator to underestimate the chew count [3].

This study has several limitations. First, we did not experiment with different food types (e.g., hard, crunchy, crispy, tough, chewy, etc.). Second, the gold standard depends on the annotators, who-although trained- are subject to mistakes and underestimation [3]. It would have been more accurate to equip the participants with piezoelectric sensors, which could capture the chewing count more accurately. Third, the length of the videos clips was one minute, which was enough time to finish the piece of food provided to the subjects. Fourth, the collected data did not include videos with different out of plane rotation (i.e., pose) or in plane rotation (i.e., orientation) as a normal chewing posture was assumed. However, the Viola-Jones algorithm can detect faces that are tilted by ±15 degrees in plane and ±45 degrees out of plane [45]. Finally, we did not perform fine-grained annotation of the chewing clips, but this can be accomplished in future works. Annotating individual chews in the videos would allow elaborate technical analysis and the development of feature-based and artificial intelligence-based counting methods.

Nonetheless, the proposed approach has several merits. First, no extra hardware is required for the deployment and usability of the counting algorithm. Once the system is installed, researchers who are interested in studying the chewing behavior of subjects (e.g., children) can use it easily. It can be used in natural everyday settings (e.g., subjects are using their smartphone or any camera-equipped smart device). Second, the study used a reasonably large number of subjects and investigated a wide range of chewing speeds. In comparison, the number of subjects in the relevant literature was less than 50 [33,35]. Third, the accuracy of the model surpasses relevant literature without requiring extra hardware or intensive computation [3,19,20,21]. Finally, the algorithm displayed robustness against different subject ages, skin colors, facial hair, or gender.

## 6. Conclusions

Chewing is an important process in the digestive system with much research dedicated to studying the effects of chew speed, chewing rate, and bolus size on the human health (e.g., BMI). In addition, it has been found that chewing speed is associated with cognitive functions.

Recent proliferation of mobile smart devices, which are equipped with cameras and strong processing power, facilitated the development of many applications from a wide range of disciplines. Another aspect to consider is the health impacts of these devices, which are being used during everyday activities including eating. Thus, the work in this paper allows for the monitoring of the chewing behavior to enable researchers to further study human dietary habits while using smart devices.

In this research, an algorithm was developed to count the number of chews from eating video recordings. The input is processed using two well-known and established methods (i.e., LPF and DWD) followed by a peak counting algorithm. Performance evaluation results greatly improved on the existing literature. Moreover, the system allows for the natural measurement without the need for expensive or uncomfortable hardware. We expect this work to enable further studies into eating and weight disorders, especially those connected to smart devices.

## Figures and Tables

**Table 1:** Annotator ICC values for the three chewing speeds.

Annotator∖Chewing Speed	Fast	Medium	Slow
All	0.91	0.94	0.96
1 & 2	0.88	0.90	0.92
2 & 3	0.83	0.90	0.95
1 & 3	0.90	0.94	0.95

**Table 2:** Performance comparison between LPF and DWD in terms of AE. SD stands for standard deviation.

Chewing Speed	LPF			DWD		
	${\mathrm{AE}}_{\mathrm{avg}}$	${\mathrm{AE}}_{\mathrm{avg}}$	±SD	${\mathrm{AE}}_{\mathrm{avg}}$	${\mathrm{AE}}_{\mathrm{avg}}$	±SD
Slow	5.42	0	4.61	5.72	0	4.8
Normal	7.47	0	6.85	7.45	0	6.85
Fast	9.84	0	9.55	10.32	0	10.42

**Table 3:** Performance comparison between LPF and DWD in terms of MAPE.

Chewing Speed	MAPE (LPF)	MAPE (DWD)
Slow	6.48%	9.09%
Normal	7.76%	7.03%
Fast	8.38%	8.31%

**Table 4:** Performance comparison between LPF and DWD in terms of RMSE.

Chewing Speed	RMSE (LPF)	RMSE (DWD)
Slow	5.56	7.64
Normal	7.93	7.09
Fast	13.03	13.43

**Table 5:** Performance comparison to the related literature.

Study	Avg Error ± SD	Counting Method	No. of Subjects
Farooq and Sazonov [3]		10.40% ± 7.03%	Peak detection in manually annotated segments	30
15.01%± 11.06%	Counting in ANN classified epochs	30
Farooq and Sazonov [22]		8.09% ± 7.16%	Piezoelectric strain sensor	5
8.26% ± 7.51%	Piezoelectric strain sensor	5
Farooq and Sazonov [42]	9.66% ± 6.28%	Linear regression of piezoelectric sensor signal	10
Bedri et al. [24]	F1-score = 90.9%	Acoustic sensor	10
Cadavid et al. [27]	Avg agreement = 93%	SVM classification of AMM spectral features	37
Taniguchi et al. [43]	Precision = 0.958	Earphone sensor	6
Wang et al. [44]	12.2%	Triaxial accelerometer on the temporalis	4
Hossain et al. [26]	Mean accuracy 88.9% ±7.4%	Deep learning and affine optical flow	28
This paper	5.42% ± 4.61 (slow) 7.47% ± 6.85 (normal) 9.84% ± 9.55 (fast)	Image processing of chewing videos	100

## Data Availability

The dataset generated during and/or analysed during the current study are available from the corresponding author on reasonable request. The dataset will be made public in a separate data article.

## Abbreviations

The following abbreviations are used in this manuscript:

BMI	Body Mass Index
EMG	Electromyography
ANN	Artificial neural networks
AMM	Active appearance model
IRB	Institutional review board
KAUH	King Abdullah University Hospital
ICC	Intra-class correlation coefficient
HF	Haar feature
ERT	ensemble of regression trees
ED	Euclidean distance
LPF	Low pass filter
DWD	Discrete wavelet decomposition
MPH	Minimum-Peak-Height
PH	Peak heights
AE	Absolute error
MAPE	Mean absolute percentage error
RMSE	Root mean squared error
